# Relationship between heart rate variability and inflammation induced by physical exercise in a sedentary healthy population

**DOI:** 10.3389/fphys.2025.1657812

**Published:** 2025-11-07

**Authors:** David Ramiro-Cortijo, Santiago Ruvira, Ricardo Alonso de Celada, Elena Muñoz-Gómez, Silvia Cañas, Jose Magalhães, Silvia M. Arribas

**Affiliations:** 1 Department of Physiology, Faculty of Medicine, Universidad Autónoma de Madrid, Madrid, Spain; 2 Food, Oxidative Stress and Cardiovascular Health (FOSCH) Research Group, Universidad Autónoma de Madrid, Madrid, Spain; 3 Institute of Food Science Research (CIAL), Universidad Autónoma de Madrid (UAM-CSIC), Madrid, Spain; 4 Department of Agricultural Chemistry and Food Science, Faculty of Science, Universidad Autónoma de Madrid, Madrid, Spain; 5 Laboratory of Metabolism and Exercise (LaMetEx), Research Centre in Physical Activity, Health and Leisure (CIAFEL), Faculty of Sport, University of Porto, Porto, Portugal

**Keywords:** exercise, healthy population, heart rate variability, sex, systemic inflammation

## Abstract

Heart rate variability (HRV) evaluates autonomic nervous system (ANS) function, and in disease conditions a reduction in HRV is associated with inflammation. We hypothesized an association between HRV and physical exercise-induced inflammation in healthy conditions. We aimed to evaluate the relationship between HRV and plasma cytokines before and after exercise, assessing the influence of sex. Sedentary young subjects (22 females, 16 males) underwent HRV evaluation through a chest band, at rest and 15 min and 48 h after a step-exercise performed until exertion, assessing R-R interval, RMSSD, low, high frequencies (LF, HF) and total power (TP). Capillary blood was obtained before and post-exercise (2 h and 48 h), assessing plasma IL-1α, IL-1β, IL-6, IL-12, MCP-1, TNFα, IL-1ra and IL-10 with multiplex ELISA. Linear regression (LRM) and additive models (GAM) were used to evaluate associations. Exercise induced LF/HF elevation (sympathetic dominance) and HF/TP reduction (parasympathetic suppression) at 15 min post-exercise, and a rise in IL-6 and IL-10 at 2 h, higher in males than in females. All measurements were normalized by 48 h. At rest, LF/HF correlated positively with IL-1β, whereas HF/TP correlated negatively with IL-1α, IL-1β, and IL-1ra; these associations persisted 15 min post-exercise, with an additional negative correlation between HF/TP and IL-12. LRM indicated a trend for an inverse relationship between HF/TP at rest and IL-1α at 2 h, and GAM revealed a nonlinear association between LF/HF at rest and IL-1β at 2 h. At 15 min post-exercise, LF/HF further associated with IL-1β and IL-12. These findings suggest that greater parasympathetic activation at rest and post-exercise may be linked to lower exercise-induced pro-inflammatory cytokines. Further research in other cohorts is warranted to confirm the capacity of HRV as indicator of inflammation in sport.

## Graphical Abstract

**Figure:**
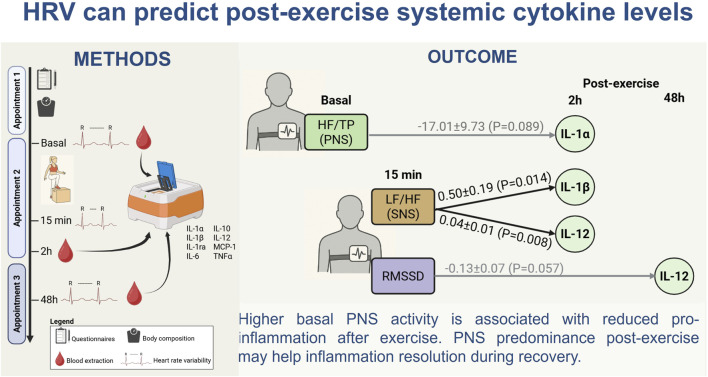

## 1 Introduction

Physical activity is fundamental for health, having well demonstrated beneficial effects on many physiological systems (Warburton and Bredin, 2017; Fernandez et al., 2018; Meyer-Lindemann et al., 2023). Despite its numerous positive actions, exercise inflicts physiological stress, eliciting muscle damage, and reducing exercise performance. The subsequent mobilization of immune cells leads to an inflammatory response, which initially participates in the spreading of inflammation, but later contributes to the recovery of muscular homeostasis. A balance between pro and anti-inflammatory cytokine networks is crucial to limit muscular damage and to resolve inflammation (Docherty et al., 2022). The magnitude of systemic cytokines in response to exercise is used as a criterion measure of internal training load in several sports (Docherty et al., 2022). However, this evaluation requires invasive methods and expensive equipment, and a practical alternative is warranted.

Heart rate variability (HRV) is the beat-by-beat fluctuation of the cardiac rhythm over a given period, which has been used to evaluate heart autonomic nervous system (ANS) function in health and disease situations. HRV can be defined by time and frequency domains. Time-domain parameters included standard deviation of normal R-R interval (SDNN) and root mean square of successive R-R interval differences (RMSSD). SDNN reflects global variability, while RMSSD is linked to short-time variability. Frequency-domain parameters encompass very low (VLF, 0.0033–0.04 Hz), low (LF, 0.04–0.15 Hz), and high (HF, 0.15–0.4 Hz) frequencies, and the sum of all frequencies comprises the total power (TP). LF is mainly due to arterial baroreflex modulation and has been linked to sympathetic nervous system (SNS) activity, although it could have a parasympathetic (PNS) component (Valenza et al., 2018), while HF primarily reflects vagal activity (Goldstein et al., 2011). To describe the level of activation of each branch of the ANS, the ratio between frequencies has been used. LF/HF ratio reflects sympatho-vagal balance; thus, an increase suggests a shift toward SNS, while a reduction indicates PNS dominance (European Society of Cardiology and North American Society of Pacing and Electrophysiology, 1996; Shaffer et al., 2014; Tarvainen et al., 2014). However, to specifically characterize PNS activation, the HF/TP ratio has been the most widely used parameter (Sassi et al., 2015).

Converging evidence suggests that ANS regulates an inflammatory reflex and HRV has been proposed to monitor human inflammatory processes (Williams et al., 2019). An association between a lower HRV, vagal tone and higher levels of pro-inflammatory cytokines has been reported in pathological situations, including panic disorders (Herhaus et al., 2023), major depression (Euteneuer et al., 2023), and psychosocial distress (Merker et al., 2022). HRV has been proposed to predict outcomes of non-communicable diseases -such as diabetes-through the relationship between HRV, vagal activity and inflammation reduction (Gidron et al., 2018; Sorski and Gidron, 2023). ANS has an important influence on physical activity; SNS is stimulated by exercise, while the PNS contributes to the recovery process. In athletes, HRV has been used as an indicator of the capacity to adapt to exercise, to evaluate the degree of activation and recovery (Bellenger et al., 2016; Güngör et al., 2024), to identify overtraining and to predict performance (Lundstrom et al., 2023). However, few studies have evaluated the association between HRV and the inflammatory response in trained individuals (Holmes et al., 2020). There is also lack of information on this relationship in non-trained subjects. Given the key role of inflammation in muscular damage induced by physical exercise it would be desirable to confirm this potential association, which could help to monitor non-invasively inflammation and recovery. The hypothesis of this study was that HRV can be used as a predictor of inflammation caused by physical exercise in non-trained subjects. Therefore, the aims were to evaluate, in a sedentary healthy young population, the relationship between HRV, and the most relevant cytokines induced by exercise, analyzing the influence of sex.

## 2 Methods

### 2.1 Study design and recruitment cohort

The present study has been approved by the Research Ethics Committee of Universidad Autónoma of Madrid (Ref. CEI-136- 2901, approved on February 9th, 2024) and the personal data is subject to the Spanish Organic Law 3/2018 of December 5th, and by the EU Regulation 2015/2283, which safeguards the security of the participant.

**FIGURE 1:**
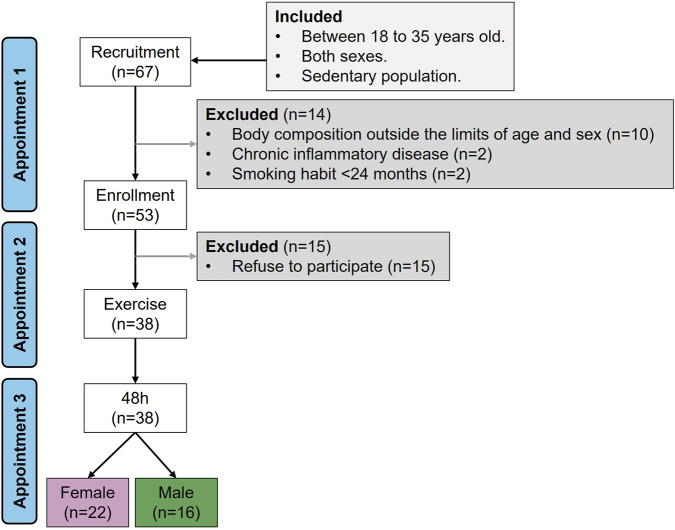
Recruitment flow chart. Sample size (n).

The participants were non-probabilistically recruited among students from the degree of Biomedical Engineering and the degree of Nursing at the Faculty of Medicine of Universidad Autónoma de Madrid (Spain). The protocol included three appointments. During appointment 1, a researcher of the team explained the protocol of the study and those interested. At this moment, the participants could resolve any type of doubt and signed the informed consent if they wished to participate in the study. Next, they filled several questionnaires, including (1) *ad hoc* sociodemographic tool related to age, educational level, economic situation and global health status, and (2) the self-report Activity Questionnaire for Adults and Adolescents (AQuAA) (Chinapaw et al., 2009), which explores the frequency and effort of common physical or sports leisure activities. The AQuAA has demonstrated validity and consistency (Busschaert et al., 2015), estimating activity by Metabolic Equivalent of Tasks (MET)*min/week (Liu et al., 2011). From AQuAA the sedentary and vigorous activities were calculated (kcal/kg*week), which was one of the exclusion criteria (see below). Thereafter, body composition was assessed by a scale-tallimeter (Tanita WB 380-H; BioLogica S.L., Barcelona, Spain) and impedanciometry (Bodystat 520, SN 510802; BioLogica S.L., Barcelona, Spain). From these data body mass index (kg/m2), fat mass (kg), lean mass (kg), muscle mass (kg), and basal metabolic rate (kcal/day) were calculated. Participants excluded from the study were those with chronic inflammatory conditions (diabetes, obesity or celiac disease), active smokers, body composition outside the normal range for age and sex, and those with a level of vigorous activities above 20 METs kcal/kg*week. A sedentary population was chosen to avoid adaptations induced by regular exercise which is known to modulate inflammation (Baskerville et al., 2024). A total of 67 participants were recruited, and from them 38 completed the study (match rate = 56.7%), 22 females and 16 males. The flow chart of recruitment and participation is shown in Figure 1.

Accepted participants were informed by e-mail and were asked to come for two additional appointments (2 and 3). They were instructed to refrain from strenuous exercise before the visits.

**FIGURE 2:**
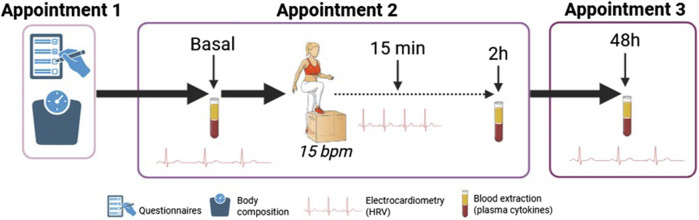
Study design. Heart rate variability (HRV), beat/minute (bpm).

During appointment 2, the participants filled in the Food Frequency Questionnaire (FFQ), a tool of 120 items that estimates an individual’s nutritional pattern (Cui et al., 2023). This tool has been widely used in epidemiological studies (Cui et al., 2021) and has been validated in Spanish population (Larroya et al., 2024). The data obtained was recorded manually in an *ad hoc* data frame and evaluated using FFQ EPIC tool for analysis (FETA) (Mulligan et al., 2014), which is an open-source software, facilitating research on dietary intake, estimating carbohydrate, protein and fat (g/day), and energy (kcal/day). Women were asked about the phase of the menstrual cycle. Thereafter, cardiovascular parameters (blood pressure and HRV) were measured before (at rest) and 15 min post-exercise. In addition, blood samples were taken before and 2 h post-exercise. Finally, the participant was asked to come back for an appointment 3 after 48 h. During this 3rd visit, cardiovascular parameters were measured, and a blood sample was taken. The study design is shown in Figure 2.

### 2.2 Heart rate variability parameters

HRV was assessed with the participant laying down on a stretcher to avoid gravitational interferences with the measurements and resting during 10 min with a Polar® band (H10, Polar Electro Ltd., Kempele, Finland) around the chest, placing the electrodes similar to V4-ECG position tilted to the left below the nipple. Polar®H10 band was coupled to the smartphone application (ELITE, https://elitehrv.com/) to register the chart measurement. ELITE estimated the time- and frequency-domain parameters. The time-domain parameters recorded were R-R interval (seconds), SDNN (in ms), and RMSSD (in ms). In addition, it was calculated as the normalized RMSSD by Ln. The frequency-domain parameters were LF, HF and TP (in ms^2^). From these parameters LF/HF and HF/TP were calculated. The HRV measurements at all time points were evaluated in the same way.

### 2.3 Blood extraction and processing

Under strict hygiene and safety measures, by personnel trained in handling biological samples, a 0.5 mL volume of capillary blood was drawn by Tasso + M20 device (TASSO Inc.; WA, United States) coupled to a lithium-heparin microtainer tube (BD Vacutainer®, NJ, United States). The device was placed on the deltoid region, previously warmed by friction and disinfected with an alcohol wipe. To avoid interference with cytokine production by food intake during the 2 h post-exercise, the participants were instructed to fast but were allowed to drink water. Immediately after extraction, the blood samples were centrifuged at 4 °C, 900 g for 10 min and plasma stored at −80 °C until used.

### 2.4 Exercise protocol

The exercise protocol consisted of up and down stepping cycles on a plyometric box with a height approximately from the participant´s knee to the ground (Vevor ES, Madrid, Spain) performed at a fixed stepping frequency of 15 cycles/min, assessed by metronome, as previously described (Larsen et al., 2007). The exercise was performed with both legs until exertion. Perceived fatigue was monitored through Borg scale (Borg, 1982). The researcher asked the volunteers about the Borg scale every 2 min and verbally encouraged them until they could not perform more repetitions. Upon the appearance of indicators of fatigue, including leg weakness, marked shortness of breath, or dizziness, the protocol was concluded. The number of complete repetitions (up and down steps) were annotated.

### 2.5 Plasma cytokines

The levels of interleukin (IL)-1α, IL-1β, IL-6, IL-12, monocyte chemoattraction protein (MCP)-1, TNFα, IL-1ra and IL-10 were simultaneously measured in 30 µL plasma using specific cartridges for these cytokines with an ELLA™ ultrasensitive multiplex system, an automated ELISA platform (Bio-techne Instruments, MN, United States), following the manufacturer’s protocol. Briefly, the plasma sample was diluted 1:2 in the diluent to load a final volume of 50 µL into the cartridge well. The ELLA™ automates simultaneous duplications via microfluidic design and micro-reactors channels, which have the detection antibody against the specific cytokine anchored. The raw signal levels detected by the instrument’s fluorescence reader (Relative Fluorescence Units, RFUs) were used to calculate analyte concentrations in pg/mL. The instrument requires for this calculation the high- and low-standards for the low and upper limits (LLOQ; ULOQ) of quantification: IL-1α (0.49; 1880 pg/mL), IL-1β (0.4; 1530 pg/mL), IL-6 (0.28; 2652 pg/mL), IL-12 (0.62; 5890 pg/mL), MCP-1 (1.52; 5780 pg/mL), TNFα (0.3; 1160 pg/mL), IL-1ra (7.37; 4500 pg/mL), IL-10 (0.38; 1446 pg/mL).

### 2.6 Statistical analysis

Statistical analysis was performed using R software (ver. 4.5.0) within RStudio interface (ver. 2025.05.0 + 496, R Foundation for Statistical Computing, Vienna, Austria) using *rio, CompareGroups, ggplot2, ggpubr, dplyr, devtools, ggcorrplot, DescTools, nlme, GGally* and *mgcv* packages. Data were expressed as median and interquartile range (IQR) [Q1; Q3] in quantitative variables and relative frequency and sample size (n) in categorical variables. For the univariate analysis, Mann–Whitney´s U test was used to prove the group differences, and for the bivariate analysis, Rho-Spearman coefficient was used to explore correlations between quantitative variables. Both the Rho-coefficient and the 95% confidence interval [95%CI] were extracted. To avoid interference with the models, missing data on cytokine variables (<10% of the total dataset) were imputed using 1/2 of the minimum value of the variable, segmented by appointment and sex.

Linear regression models (LRM) were constructed considering the cytokine level as the dependent variable and HRV parameters as independent variables. The association between dependent and independent variables as a function of time was built step-by-step following the significance of the bivariate analysis. In addition, LRM were adjusted by significant variables in the univariate analysis. From LRM, the standardized coefficients (β) and standard errors of each factor were extracted. Furthermore, considering that the temporal relationship in systemic cytokines could have a non-linear relationship that masked LRM, general additive models (GAM) were built considering the smoothed HRV term. The GAM models were adjusted for the same variables as the LRM models. From GAM, the effective degrees of freedom (EDF) value were extracted, which estimates whether the relationship between variables can be non-linearly dependent. To compare LRM and GAM quality and fitness, the Akaike Information Criterion (AIC) criterion was used. Generally, a lower AIC score indicates a better-fit model. Statistical significance was assumed for P-values (P) <0.05.

## 3 Results

### 3.1 Cohort characteristics

At appointment 2, 18.2% (n = 4) of recruited women were in active menstruation, 45.5% (n = 10) were in the follicular phase, and 36.4% (n = 8) were in the luteal phase of the menstrual cycle. Regarding sex differences in sociodemographic, nutritional and anthropometric factors, significant differences were found in age, which was larger in females compared to males, and in protein intake, which was higher in male than female participants. Body composition was also different between sexes, as expected. The cohort had a sedentary-to-light intensity aerobic physical activity, with non-significant differences between sexes in weekly sedentary or vigorous activities (Table 1).

**TABLE 1:** Nutritional pattern, body composition and physical activity of the cohort.

Variables	Total (n = 38)	Female (n = 22)	Male (n = 16)	*P*
Age (years)	20.0 [18.0; 20.0]	20.0 [19.0; 21.0]	18.0 [18.0; 20.0]	0.006
Daily meals	3.5 [3.0; 4.0]	3.5 [3.0; 4.0]	3.5 [3.0; 4.0]	0.606
Carbohydrate intake (g/day)	194 [148; 235]	195 [154; 235]	174 [147; 234]	0.554
Protein intake (g/day)	89.4 [76.3; 113]	84.1 [75.8; 93.0]	103 [86.9; 129]	0.038
Fat intake (g/day)	66.1 [58.3; 89.7]	61.7 [57.6; 82.0]	76.0 [65.4; 111]	0.117
Energy intake (kcal/day)	1672 [1494; 2136]	1649 [1423; 1898]	1764 [1567; 2520]	0.425
Body mass index (kg/m2)	21.7 [20.2; 23.5]	21.6 [19.2; 22.9]	21.7 [20.2; 23.6]	0.871
Fat mass (kg)	12.5 [10.8; 15.8]	15.2 [12.9; 17.0]	10.4 [8.85; 11.2]	<0.001
Lean mass (kg)	44.2 [40.0; 54.4]	40.3 [38.5; 42.9]	55.4 [53.0; 58.7]	<0.001
Muscle mass (kg)	20.1 [18.3; 27.6]	18.5 [17.8; 19.2]	28.1 [26.7; 28.7]	<0.001
Basal metabolic rate (kcal/day)	1490 [1388; 1698]	1397 [1352; 1460]	1722 [1656; 1815]	<0.001
Sedentary activities (kcal/kg*week)	10.4 [4.71; 17.2]	12.4 [5.62; 17.9]	6.11 [4.46; 11.4]	0.249
Vigorous activities (kcal/kg*week)	2.09 [0.35; 4.57]	2.32 [0.93; 4.74]	0.85 [0.15; 4.12]	0.320

Data shows median and interquartile range [Q1; Q3]. Physical activity was calculated using the self-report Activity Questionnaire for Adults and Adolescents (AQuAA) as weekly metabolic equivalents of task (METs) by kg. The P-value (P) was extracted from the non-paired Mann-Whitney U test, sample size (n).

### 3.2 Exercise performance

The number of step repetitions were significantly higher in male (634 [410; 982]) compared to female participants (374 [296; 455]; P = 0.036). When the number of repetitions were normalized for muscle mass, no significant differences were found (female = 20.4 [16.2; 23.9] rep/kg muscle, male = 22.8 [14.1; 35.6] rep/kg muscle; P = 0.790).

### 3.3 Heart rate variability

**FIGURE 3:**
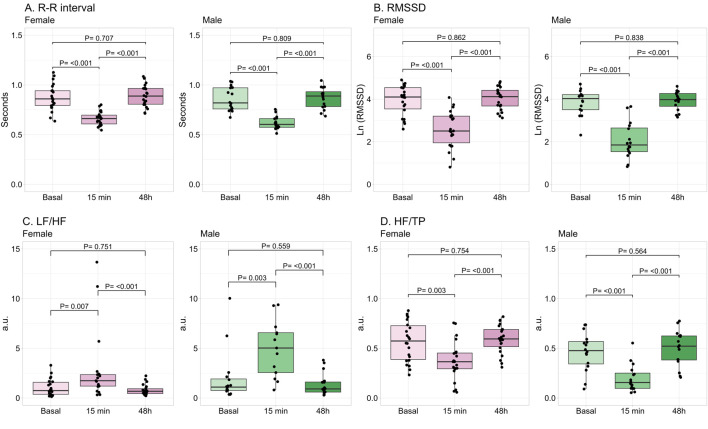
Changes in time- and frequency-domains of heart rate variability by exercise. Data shows median and interquartile range [Q1; Q3]. The P-value (P) was extracted from the non-paired Mann-Whitney U test. Root mean square of successive R-R interval differences (RMSSD), Low frequency (LF), high frequency (HF), total power (TP), arbitrary units (a.u.), female = 22, male = 16.

All HRV variables were significantly modified at 15 min post-exercise. The time-domain variables (R-R interval and RMSSD) were reduced (Figures 3A,B). Regarding frequency-domain, LF/HF was increased, while HF/TP was significantly reduced (Figures 3C,D). These modifications were observed in both female and male participants, returning to basal values after 48 h in both sexes.

Regarding the influence of sex, in basal conditions no significant differences were found between female and male participants in any of the HRV parameters. Absolute variables of HRV were described in Supplementary Table S1. At 15 min post-exercise, females had significantly lower mean heart rate and LF/HF, while HF/TP was higher than males (Table 2). When these parameters were normalized by the number of repetitions, HR and LF/HF were not significantly different between sexes (HR: P = 0.124; LF/HF: P = 0.132). However, HF/TP remained significantly larger in females compared to males (P = 0.001). At 48 h, the parameters were not statistically different between sexes (Table 2).

**TABLE 2:** Time- and frequency-domains of heart rate variability by sex in each appointment.

Appointment	Variables	Female (n = 22)	Male (n = 16)	*P*
Appointment 2 (Basal)	Mean heart rate (bpm)	76.0 [69.0; 84.5]	76.5 [68.8; 84.5]	0.953
	R-R interval (sec)	0.86 [0.79; 0.94]	0.82 [0.76; 0.97]	0.564
	RMSSD (Ln (RMSSD))	4.10 [3.54; 4.54]	4.03 [3.51; 4.22]	0.626
	LF/HF (a.u.)	0.75 [0.37; 1.58]	1.10 [0.76; 1.92]	0.160
	HF/TP (a.u.)	0.57 [0.39; 0.73]	0.48 [0.34; 0.57]	0.165
Appointment 2 (15 min)	Mean heart rate (bpm)	103 [96.2; 109]	118 [111; 122]	0.001
	R-R interval (sec)	0.66 [0.61; 0.70]	0.60 [0.57; 0.66]	0.057
	RMSSD (Ln (RMSSD))	2.51 [1.95; 3.20]	1.85 [1.54; 2.65]	0.070
	LF/HF (a.u.)	1.74 [1.21; 2.40]	6.15 [3.00; 9.32]	0.004
	HF/TP (a.u.)	0.37 [0.29; 0.45]	0.16 [0.10; 0.25]	0.005
Appointment 3 (48 h)	Mean heart rate (bpm)	73.5 [65.0; 78.5]	70.0 [67.8; 81.0]	0.722
	R-R interval (sec)	0.89 [0.81; 0.97]	0.89 [0.78; 0.93]	0.615
	RMSSD (Ln (RMSSD))	4.12 [3.68; 4.42]	3.98 [3.67; 4.27]	0.554
	LF/HF (a.u.)	0.68 [0.45; 0.93]	0.92 [0.60; 1.61]	0.135
	HF/TP (a.u.)	0.59 [0.52; 0.69]	0.52 [0.38; 0.62]	0.132

Data shows median and interquartile range [Q1; Q3]. The P-value (P) was extracted from the non-paired Mann-Whitney U test. Beat/minute (bpm), Root mean square of successive R-R interval differences (RMSSD), Low frequency (LF), high frequency (HF), total power (TP), arbitrary units (a.u.), sample size (n).

### 3.4 Plasma cytokine levels

**FIGURE 4:**
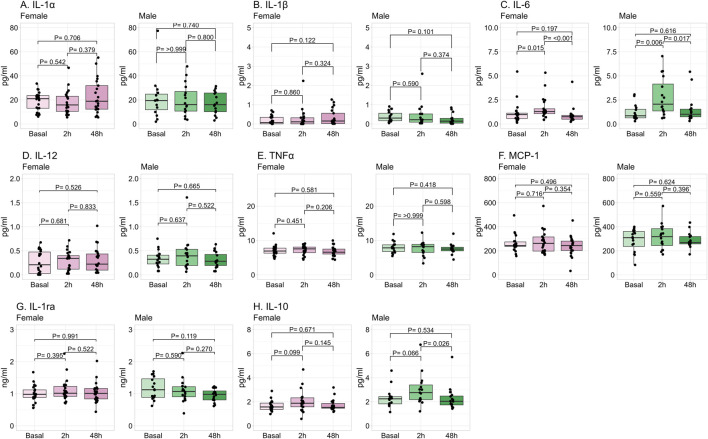
Progression in plasma cytokine levels by exercise. Data shows median and interquartile range [Q1; Q3]. The P-value (P) was extracted from the non-paired Mann-Whitney U test. Interleukin (IL), monocyte chemoattractant protein (MCP), tumor necrosis factor (TNF), female = 22, male = 16.

The progression in plasma cytokines induced by exercise is shown in Figure 4. IL-6 was significantly increased at 2 h post-exercise in both sexes, retuning to basal levels at 48 h. IL-10 was significantly increased at 2 h in males, and in females it tended to increase without reaching statistical significance. Both IL-6 and IL-10 returned to basal levels at 48 h in both sexes. The other cytokines were not significantly modified by exercise.

A positive significant correlation was found between the number of repetitions and IL-6 (Rho = 0.48 95%CI [0.19, 0.70]; P = 0.002), MCP-1 (Rho = 0.60 95%CI [0.35; 0.77]; P < 0.001), and IL-10 (Rho = 0.39 95%CI [0.09; 0.63]; P = 0.014) measured at 2 h post-exercise, while there was no correlation with cytokine levels 48 h post-exercise (data not shown).

Regarding the influence of sex on cytokines, no significant differences were found in the cytokine levels in basal conditions. At 2 h post-exercise, IL-1β and IL-10 were significantly higher in male compared to female participants, and IL-6 also tended to be larger. At 48 h post-exercise, MCP-1 and IL-10 were significantly higher in males compared to females, with the same tendency for TNFα and IL-6 (Table 3).

**TABLE 3:** Plasma cytokines levels by sex in each appointment.

Appointment	Variables	Female (n = 22)	Male (n = 16)	*P*
Appointment 2 (Basal)	IL-1α (pg/mL)	21.0 [13.1; 23.6]	19.6 [12.8; 26.6]	0.941
	IL-1β (pg/mL)	0.07 [0.01; 0.34]	0.36 [0.17; 0.64]	0.221
	IL-6 (pg/mL)	0.98 [0.57; 1.12]	0.85 [0.64; 1.52]	0.881
	IL-12 (pg/mL)	0.21 [0.03; 0.48]	0.33 [0.23; 0.41]	0.314
	TNFα (pg/mL)	6.94 [6.32; 7.80]	7.88 [6.92; 9.08]	0.235
	MCP-1 (pg/mL)	244 [237; 274]	310 [236; 362]	0.107
	IL-1ra (pg/mL)	982 [888; 1121]	1126 [890; 1465]	0.274
	IL-10 (pg/mL)	1.58 [1.38; 1.96]	2.24 [1.83; 2.42]	0.758
Appointment 2 (2 h)	IL-1α (pg/mL)	15.8 [10.5; 23.1]	16.1 [10.7; 27.0]	0.756
	IL-1β (pg/mL)	0.11 [0.01; 0.32]	0.22 [0.10; 0.59]	0.044
	IL-6 (pg/mL)	1.30 [1.11; 2.12]	2.06 [1.35; 4.15]	0.084
	IL-12 (pg/mL)	0.34 [0.12; 0.40]	0.40 [0.20; 0.53]	0.178
	TNFα (pg/mL)	7.60 [6.52; 8.22]	8.22 [6.54; 8.88]	0.359
	MCP-1 (pg/mL)	262 [198; 316]	318 [244; 384]	0.121
	IL-1ra (pg/mL)	1016 [919; 1228]	1072 [906; 1219]	0.825
	IL-10 (pg/mL)	1.92 [1.58; 2.35]	2.88 [2.18; 3.62]	0.009
Appointment 3 (48 h)	IL-1α (pg/mL)	19.1 [12.5; 34.6]	16.9 [10.4; 26.6]	0.442
	IL-1β (pg/mL)	0.16 [0.02; 0.66]	0.15 [0.04; 0.29]	0.573
	IL-6 (pg/mL)	0.76 [0.49; 0.90]	1.01 [0.75; 1.54]	0.063
	IL-12 (pg/mL)	0.22 [0.10; 0.44]	0.28 [0.20; 0.42]	0.574
	TNFα (pg/mL)	6.61 [6.08; 7.51]	7.52 [7.00; 8.05]	0.092
	MCP-1 (pg/mL)	244 [203; 281]	268 [258; 319]	0.038
	IL-1ra (pg/mL)	1012 [839; 1164]	982 [809; 1088]	0.605
	IL-10 (pg/mL)	1.54 [1.46; 1.86]	2.04 [1.74; 2.48]	0.008

Data shows median and interquartile range [Q1; Q3]. The P-value (P) was extracted from the non-paired Mann-Whitney U test. Interleukin (IL), monocyte chemoattractant protein (MCP), tumor necrosis factor (TNF), sample size (n).

At baseline and 2 h post-exercise, IL-1β, but not IL-1α, showed a positive correlation with IL-1ra (basal: Rho = 0.38 95%CI [0.07; 0.62], P = 0.020; 2 h: Rho = 0.41 95%CI [0.10; 0.64], P = 0.012). At 48 h post-exercise, neither of these cytokines was correlated.

### 3.5 Correlations between HRV and exercise-induced cytokine progression

**FIGURE 5:**
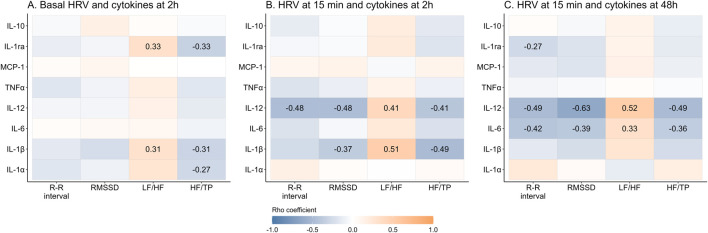
Correlation between time- and frequency-domains of the heart rate variability and plasma cytokines before and after exercise. Data shows Rho’s coefficient. The P-value (P) was extracted from Spearman correlation. Significant coefficients are shown in gradient color. Interleukin (IL), monocyte chemoattractant protein (MCP), tumor necrosis factor (TNF), Root mean square of successive R-R interval differences (RMSSD), Low frequency (LF), high frequency (HF), total power (TP).

Regarding the correlations between basal HRV and cytokines 2 h post-exercise (which represent the immediate pro-inflammatory period) a positive significant correlation was found between LF/HF, and IL-1β and IL-1ra. Instead, negative and significant correlations were found between HF/TP with IL-1α, IL-1β and IL-1ra (Figure 5A). With respect to HRV at 15 min post-exercise and cytokines measured during the immediate proinflammatory period, a positive association was found between LF/HF and IL-1β and IL-12, while HF/TP and RMSSD negatively correlated with these cytokines. Additionally, R-R interval showed a significant negative correlation with IL-12 (Figure 5B). Regarding the correlations between HRV variables at 15 min and cytokines at 48 h post-exercise (which represents the resolution period), a similar pattern of correlations was found, a positive correlation between LF/HF and IL-12 and IL-6, and negative for R-R interval, RMSSD and HF/TP with both cytokines. Additionally, R-R interval was negatively correlated with IL-1ra (Figure 5C). There were no significant correlations between basal HRV and plasma cytokines at basal point or 48 h post-exercise. Similarly, no correlations were found between HRV parameters at 48 h and previous plasma cytokines (data not shown).

### 3.6 Association between HRV and exercise-induced cytokine progression

The associations between HRV parameters and cytokines were explored through Linear and General Additive Regression Models (LRM and GAM). Regarding the influence of HRV in basal conditions on cytokine production, according to the LRM, higher HF/TP was associated with a trend towards lower IL-1α levels 2 h post-exercise, while other HRV variables showed no significant associations. Notably, the GAM revealed that basal LF/HF had a significant nonlinear relationship with IL-1β (Table 4; Supplementary Figure S1).

**TABLE 4:** Linear and additive analysis of HRV and plasma cytokine over time induced by physical activity.

Basal HRV and cytokines at 2 h		β±SE	EDF	AIC
IL-1α	HF/TP	−17.01 ± 9.73 (P = 0.089)	1.30 (P = 0.146)	LRM = 299.4 GAM = 299.1
IL-1β	LF/HF	0.80 ± 0.50 (P = 0.118)	6.24 (P < 0.001)	LRM = 209.8 GAM = 75.1
	HF/TP	−0.69 ± 4.57 (P = 0.882)	1.0 (P = 0.988)	
IL-1ra	LF/HF	−17.52 ± 54.25 (P = 0.749)	1.0 (P = 0.749)	LRM = 565.9 GAM = 565.9
	HF/TP	−567.9 ± 495.8 (P = 0.260)	1.0 (P = 0.260)	
HRV at 15 min and cytokines at 2 h		β±SE	EDF	AIC
IL-1β	RMSSD	0.67 ± 1.02 (P = 0.515)	1.0 (P = 0.847)	LRM = 209.5 GAM = 182.5
	LF/HF	0.50 ± 0.19 (P = 0.014)	6.71 (P < 0.001)	
	HF/TP	1.15 ± 5.26 (P = 0.828)	1.14 (P = 0.857)	
IL-12	R-R interval	−0.84 ± 0.93 (P = 0.372)	1.53 (P = 0.217)	LRM = 7.52 GAM = −9.53
	RMSSD	−0.05 ± 0.09 (P = 0.567)	1.0 (P = 0.795)	
	LF/HF	0.04 ± 0.01 (P = 0.008)	5.71 (P = 0.008)	
	HF/TP	0.45 ± 0.38 (P = 0.249)	3.45 (P = 0.512)	
HRV at 15 min and cytokines at 48 h		β±SE	EDF	AIC
IL-6	R-R interval	−2.99 ± 4.69 (P = 0.530)	4.32 (P = 0.150)	LRM = 130.8 GAM = 53.7
	RMSSD	0.09 ± 0.44 (P = 0.831)	9.0 (P = 0.004)	
	LF/HF	0.02 ± 0.07 (P = 0.757)	8.31 (P < 0.001)	
	HF/TP	−0.93 ± 1.93 (P = 0.757)	2.29 (P = 0.013)	
IL-12	R-R interval	−0.13 ± 0.71 (P = 0.855)	1.0 (P = 0.755)	LRM = −12.6 GAM = −14.2
	RMSSD	−0.13 ± 0.07 (P = 0.057)	1.0 (P = 0.049)	
	LF/HF	0.02 ± 0.01 (P = 0.088)	2.61 (P = 0.187)	
	HF/TP	0.31 ± 0.29 (P = 0.305)	1.22 (P = 0.907)	
IL-1ra	R-R interval	−665.7 ± 665.5 (P = 0.324)	1.0 (P = 0.324)	LRM = 540.2 GAM = 540.2

The models were adjusted by age, sex, protein intake and muscle mass. Estimated Degrees of Freedom (EDF); Akaike Information Criterion (AIC). In the linear regression model (LRM) was extracted standardized coefficients (β) ± standard error (SE). In the generalized additive model (GAM), the smoothed term of the model was the variable HRV., Interleukin (IL), Root means square of successive R-R interval differences (RMSSD), Low frequency (LF), high frequency (HF), total power (TP).

With respect to the influence of HRV 15 min post-exercise on cytokine production, according to the LRM, an increased LF/HF ratio was linked to elevated plasma IL-1β and IL-12 levels 2 h post-exercise. GAM revealed that basal LF/HF had a significant nonlinear relationship with both cytokines (Table 4; Supplementary Figure S1).

When examining HRV at 15 min as a predictor of cytokine levels at 48 h, significant nonlinear associations were identified between RMSSD, LF/HF and HF/TP ratios and IL-6. Additionally, in the LRM, RMSSD demonstrated a negative trend to associate with IL-12 levels, reaching significance in the GAM, while LF/HF exhibited a positive trend towards significance (Table 4; Supplementary Figure S1).

## 4 Discussion

**FIGURE 6:**
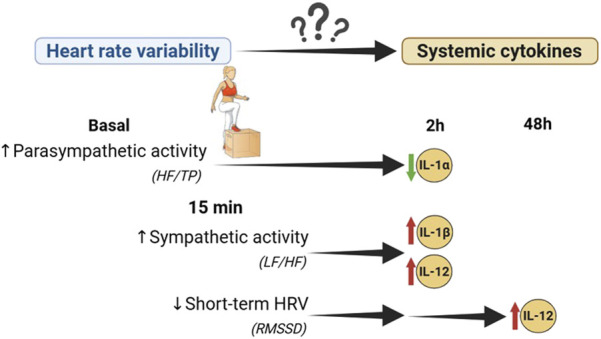
Association between heart rate variability and inflammation induced by physical exercise. Interleukin (IL), Root means square of successive R-R interval differences (RMSSD), Low frequency (LF), high frequency (HF), total power (TP).

HRV monitoring can evaluate ANS function in physiological and pathological scenarios. The use of portable devices and smartphone applications to measure HRV has popularized its use in sport as marker of exercise load, to individualize training (Stanley et al., 2013). In this context, inflammation is also a relevant issue, but its monitoring is time consuming and requires invasive methods. Given the relationship between ANS and inflammation demonstrated in several pathologies, the objective of the present study was to evaluate this relationship in the context of exercise in a healthy population, to assess the capacity of HRV as predictor of inflammation. Through regression models our study demonstrates that larger PNS dominance in basal conditions and lower SNS activation by exercise associated with a lower level of pro-inflammatory cytokines induced in the immediate inflammatory period, namely, those related to IL-1 family and IL-12 (Figure 6). These results could support the influence of vagal activation to modulate inflammation induced by exercise.

### 4.1 Effect of exercise on cytokine profile

Physical exercise inflicts skeletal muscle damage, which is followed by an inflammatory response (Lee et al., 2017). This process can be monitored by the systemic levels of cytokines (Małkowska and Sawczuk, 2023). In the population under study, the step-exercise protocol elevated IL-6, one of the most relevant cytokines in exercise physiology, which is produced by skeletal muscle and other cell types (Małkowska and Sawczuk, 2023). We did not find differences in other pro-inflammatory cytokines such as TNFα, MCP-1 or IL-1 family, also pivotal in the context of sport. Since these molecules do not show a significant elevation in moderate exercise (Docherty et al., 2022), we suggest that in our population, although the exercise was performed until exertion, the intensity level was moderate. Perceived exhaustion in non-athletes occurs a lower percentage of VO_2_max compared to trained counterparts (Travlos and Marisi, 1996) and perceived effort and physiological intensity do not always correspond to real physiological stress (Pageaux and Lepers, 2016). The positive correlation between the number of repetitions and MCP-1 supports our assumption. The elevation of TNFα, MCP-1 and IL-1 has been reported after prolonged or strenuous exercise, such as marathons (Ostrowski et al., 1999; Maciejewska-Skrendo et al., 2022), although to a much lower extent compared to IL-6 (Małkowska and Sawczuk, 2023).

We found a concomitant increase of IL-6 and IL-10 at 2 h post-exercise. This agrees with the literature showing that IL-6 released as myokine can contribute to inflammation resolution stimulating anti-inflammatory cytokines such as IL-10 and IL-1ra (Pedersen and Febbraio, 2008; Docherty et al., 2022) and inhibiting TNFα and IL-1 family (Ringleb et al., 2024). We did not find an elevation of IL-1ra, which blocks the effect of IL-1 family (Weber et al., 2010; Docherty et al., 2022), likely related to the low level of these pro-inflammatory cytokines released in response to exercise in our cohort, since a correlation was found between IL-1ra and IL-1β was found. The cytokine changes in the present study were observed at 2 h post-exercise returning to baseline after 48 h, with a similar time-course of the above-mentioned studies.

Regarding the influence of sex on plasma cytokines, we found no significant differences in basal conditions. However, after exercise, smaller IL-6 levels were detected in females. We suggest that this is likely related to the lower number of repetitions performed, since IL-6 release is related to the duration and intensity of exercise (Baskerville et al., 2024). The menstrual cycle phase and systemic sex hormones may also have an influence on this cytokine (Barba-Moreno et al., 2022; Baskerville et al., 2024). Although we evaluated the ovarian cycle in our population, we did not analyze differences between phases due to small sample size. This aspect deserves further analysis with a larger population and assessing cytokines together with sex hormones at different ovarian cycle points.

We also found that female had lower elevation of IL-10 compared to male participants, in agreement with a report on intramuscular cytokine mRNA expression induced by exercise (Luk et al., 2021). It has also been described that IL-10-mediated inhibition of proinflammatory cytokines (TNFα, IL-1β, and IL-15) is lower in females, suggesting a larger recovery period after exercise for women (Islam et al., 2022). In summary, we conclude that the exercise used in the present study induces a moderate inflammatory response through IL-6, followed by a physiological anti-inflammatory effect mediated by IL-10. The lack of significant elevation of other cytokines may be related to the moderate exercise protocol, together with the negative feed-back loops created by IL-6 and IL-10 and, although not significantly elevated, may be relevant and therefore, their relationship with HRV was also explored.

### 4.2 Relationship between HRV and exercise

Classically, HRV has been a reliable marker to explore the cardiac ANS function. The standards of measurement and physiological interpretation was established in 1996 by the task Force of the European Society of Cardiology and the North American Society of Pacing and Electrophysiology (European Society of Cardiology and North American Society of Pacing and Electrophysiology, 1996). Since then, HRV assessment has been used in a broad range of applications in health and disease, including exercise and training prescriptions. New HRV methodologies and their application have been described more recently, but overall traditional time and frequency-domain HRV parameters remain the methods of choice for ANS assessment (Sassi et al., 2015). HRV can be evaluated through an ECG. However, for the collection of data during physical activity, chest strap units coupled with smartphones are useful validated devices, being most accurate when assessing locomotor activities with repetitive movements (Mühlen et al., 2021), such as the exercise used in the present study. The device used, Polar® H10 band device has these characteristics and can register R-R duration of ECG and by validating ELITE HRV software can be determined the classical time- and frequency-domain parameters (Tarvainen et al., 2014).

As expected, exercise induced an activation of SNS, shown by the increase in heart rate, reduction in R-R intervals and RMSSD time-dependent parameters, and by the rise in LF/HF and reduction of HF/TP frequency-domain parameters, which also indicate sympathetic branch predominance (Docherty et al., 2022). While LF/HF has been proposed that it is not suitable for the evaluation for the SNS activity during prolonged exercise or in highly trained population with adaptations (Hayano and Yuda, 2021), this parameter can be used as indicator of SNS modulation in sedentary population (Ernst, 2017). HRV parameters returned to baseline at 48 h, due to the shift back to PNS dominance during recovery (Michael et al., 2017). We did not find significant differences between sexes in basal conditions, even though several studies have found that females have a lower HRV compared to males (Docherty et al., 2022). In a young healthy population similar HRV values than those reported in the present study were found, but it was demonstrated a significantly lower LF/HF at rest in female participants (Bohn et al., 2024). We suggest that the lack of differences in our study may be related to the effect of sex-hormones that influences on HRV (Von Holzen et al., 2016; Ramesh et al., 2022), and we have female participants in differences menstrual cycle phase. Despite lack of sex differences at rest, we found that exercise induced less SNP activation in female compared to male counterparts, evidenced by both time- and frequency-domain parameters. We think this can be related to the lesser volume of exercise performed by the female participants, since it has been reported that exercise intensity is directly proportional to SNS activation and lower HRV, at least at moderate intensities (Achten and Jeukendrup, 2003; Michael et al., 2017), like that in the present study. In the same line, a metanalysis also demonstrated that, in general, female show a greater vagal activity, which may contribute to their better cardiovascular health during fertile life (Koenig and Thayer, 2016). Among potential mechanisms the influence of estrogens has been proposed, since in rodents ovariectomy and vagotomy reversed these effects in females (Du et al., 2006). Oxytocin has also suggested playing a role, through the synapse of oxytocin-type neurons on the nucleus of the solitary tract, increasing the vagal outflow (Higa et al., 2002).

The relationship between HRV and inflammation has been put forward in clinical settings, establishing that vagus nerve can inhibit inflammation by neuroendocrine and neuroimmunological reflexes (Alen, 2022). HRV and inflammation are relevant aspects to monitor exercise, avoiding overtraining and maladaptive states, Therefore, the present study aimed to establish the relationship between HRV parameters and cytokines released by exercise. Even though IL-6 was cytokine showing larger elevation in our experimental setting, we did not find a relationship with HRV. Similarly, in trained individuals performing resistance anaerobic activity, no relationship was found between IL-6 and short-term HRV by RMSSD, although negative correlations were found post-exercise with lactate (Holmes et al., 2020). Instead, several correlations were found between basal HRV time and frequency-domain parameters and IL-1 family 2 h post-exercise. These correlations indicate a link between a lower PNS branch dominance in basal conditions and these pro-inflammatory cytokines. Similar correlations were found between HRV parameters evaluated after exercise and cytokine levels in the immediate inflammatory period 2 h post-exercise. These data are in line with the findings that individuals with a reduced parasympathetic tone may exhibit more pronounced inflammatory response to exercise, including elevation of IL-1β levels (Moldoveanu et al., 2001). These correlations were confirmed by regression models, and adjusted LRM found an association between higher basal PNS activity and a tendency to decrease IL-1α at 2 h after physical activity. Furthermore, PNS activity at 15 min post-exercise was associated with the reduction of IL-6 and the increase of IL-1ra at 48 h, which represents the recovery period, suggesting that higher PNS activation may also be beneficial in the resolution of inflammation. We also found that additive models demonstrated a strong nonlinear and complex association between ANS activation and systemic cytokine levels. We propose that this may be related to the dual pro- and anti-inflammatory effects of exercise and complex feed-back loops between cytokines induced by exercise (Baskerville et al., 2024). Our data suggests a potential association between HRV parameters and cytokine production in response to exercise, which could be relevant in the field of sport physiology and training.

### 4.3 Limitations and future perspectives

The present study has some limitations. Firstly, the results should be interpreted with caution, as the statistical significance may be affected by potential false positives given that no correction for multiple comparisons was applied. Moreover, although the sample size could be considered large and the study is exploratory in nature, a formal sample size calculation was not performed, which may limit the strength of the statistical inferences. On the other hand, the lack of assessment of impact of menstrual cycle on the HRV and inflammation variables could be another limitation. Future studies focused on these aspects monitoring HRV, cytokines and sex-hormones would shed light on this important aspect, particularly in the field of female competition sport. In addition, our study only explores the humoral aspect of inflammation induced by exercise, and it would be interesting to evaluate the cellular response. Furthermore, the cytokines values below the detection limit were imputed using a simple substitution method (½ of the minimum observed value for the variable). It is known that this approach can introduce bias and underestimate variability. However, given that the proportion of imputed values was below 10% and the analysis was exploratory in nature, this method could be considered acceptable (Chen et al., 2011). Nevertheless, more advanced approaches such as multiple imputations, which better preserve distributional properties and propagate uncertainty, should be considered in future studies—particularly when the primary outcome involves diagnostic applications of HRV in relation to exercise-induced cytokine responses. Finally, the present study reveals an important relationship between HRV and inflammation in a sedentary healthy young cohort, being interesting to evaluate the meaning of the present data in other populations, such as menopausal women or athletes to determine the value of these findings in these contexts.

## 5 Conclusion

Our findings indicate a potential link between HRV parameters and exercise-induced cytokine responses, highlighting their possible relevance as non-invasive markers in sports physiology and training monitoring. The present study demonstrates that a higher basal PNS activity is linked to a lower release of pro-inflammatory cytokines in the active inflammatory period post-exercise. PNS predominance immediately after exercise may also be beneficial in the resolution of inflammation in the recovery period. This study also evidences a strong nonlinear and complex association between ANS activation and systemic cytokines. Importantly, sex differences in HRV and exercise-induces inflammation which require a further in-depth study.

## Data Availability

The raw data supporting the conclusions of this article will be made available by the authors, without undue reservation.
