# Supplementary material for: Relationship between heart rate variability and inflammation induced by physical exercise in a sedentary healthy population
Source: Front Physiol. 2025 Nov 7;16:1657812. doi: 10.3389/fphys.2025.1657812 (PMC12634326; doi:10.3389/fphys.2025.1657812)

**Table S1.** The absolute value of time- and frequency-domains of heart rate variability by sex in each appointment.

|  |  | Female (n=22) | Male (n=16) | *P* |
| --- | --- | --- | --- | --- |
| Appointment 2 (Basal) | SDNN (ms) | 70.3 [43.6; 93.7] | 67.8 [56.8; 82.3] | 0.802 |
|  | RMSSD (ms) | 60.5 [34.7; 93.7] | 56.5 [33.3; 67.8] | 0.626 |
|  | Low frequency (ms^2^) | 1041 [362; 2356] | 1233 [791; 1966] | 0.525 |
|  | High frequency (ms^2^) | 1870 [576; 4096] | 1262 [591; 2636] | 0.337 |
| Appointment 2 (15 min) | SDNN (ms) | 28.8 [20.4; 36.9] | 24.3 [17.6; 30.3] | 0.198 |
|  | RMSSD (ms) | 12.3 [7.19; 24.7] | 6.38 [4.65; 14.2] | 0.067 |
|  | Low frequency (ms^2^) | 141 [63.2; 220] | 84.7 [65.1; 238] | 0.359 |
|  | High frequency (ms^2^) | 79.9 [27.6; 202] | 20.5 [8.05; 62.6] | 0.058 |
| Appointment 3 (48h) | SDNN (ms) | 60.3 [48.0; 90.8] | 65.9 [55.2; 75.1] | 0.723 |
|  | RMSSD (ms) | 61.5 [39.7; 82.9] | 53.5 [39.3; 71.3] | 0.554 |
|  | Low frequency (ms^2^) | 774 [581; 1618] | 1087 [732; 1701] | 0.478 |
|  | High frequency (ms^2^) | 1472 [630; 2889] | 1198 [550; 1622] | 0.375 |

Data shows median and interquartile range [Q1; Q3]. The P-value (P) was extracted from the non-paired Mann-Whitney U test. Standard Deviation of Normal-to-Normal interval (SDNN), Root mean square of successive R-R interval differences (RMSSD), sample size (n).

**Figure S1.** Additive analysis plots of the association between HRV parameters and cytokines. Root mean square of successive R-R interval differences (RMSSD), Low frequency (LF), high frequency (HF), total power (TP), Interleukin (IL).


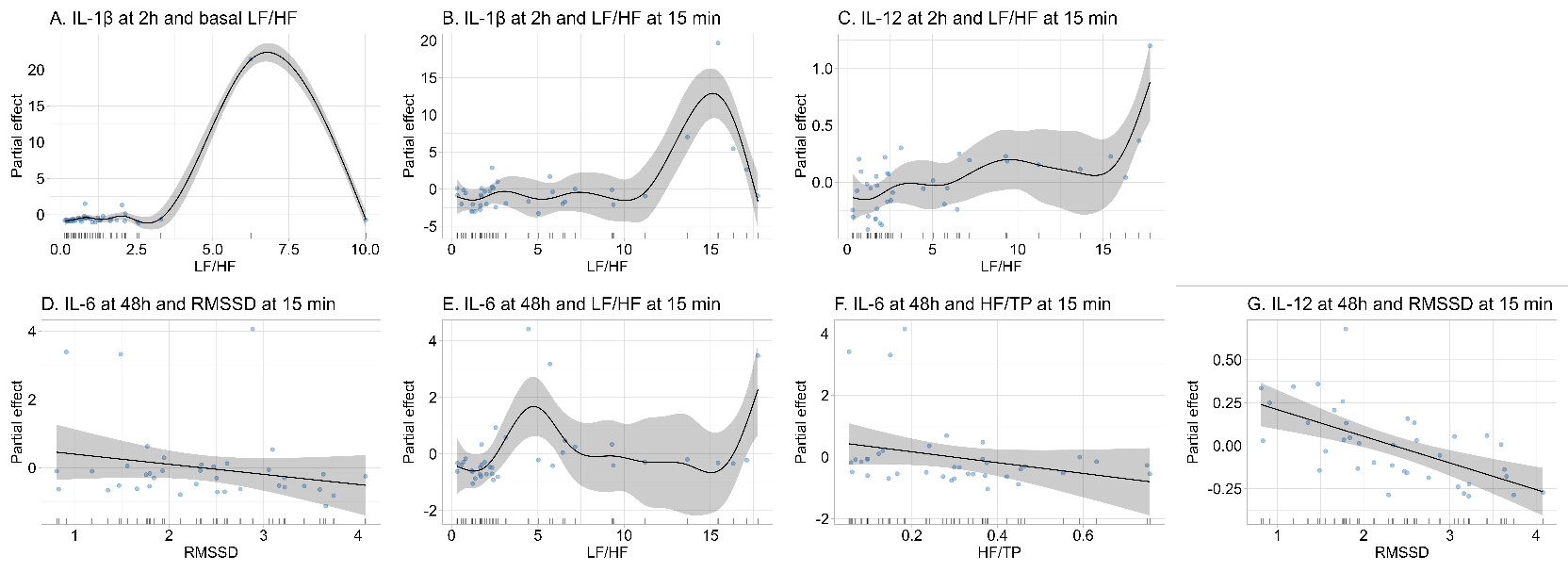

Supplement: Supplementary file 1 [file DataSheet1.docx]
